# Genome analysis screening virulence genes for the altered pathogenicity of *Riemerella anatipestifer* in hens

**DOI:** 10.3389/fmicb.2025.1705927

**Published:** 2025-12-10

**Authors:** Junfeng Lv, Hui Chen, Xiuli Ma, Yang Cong, Xiaofei Song, Yufeng Li, Yuehua Gao, Zhuoming Qin

**Affiliations:** 1 Poultry Institute, Shandong Academy of Agricultural Sciences, Jinan, China; 2 Shandong Provincial Key Laboratory of Livestock and Poultry Breeding, Jinan, China; 3 Qilu Animal Health Products Co., Ltd., Jinan, China

**Keywords:** *Riemerella anatipestifer*, laying hens, genome analysis, virulence genes, pathogenicity alteration

## Abstract

**Background:**

In recent years, *Riemerella anatipestifer* infection in chickens has markedly increased, resulting in substantial economic losses to the poultry industry. The present study was designed to assess the pathogenicity of *R. anatipestifer* in laying hens and to elucidate the molecular mechanisms underlying its altered virulence.

**Methods:**

*Riemerella anatipestifer* strains were isolated from laying hens presenting with oviduct obstruction and diminished egg production. Animal challenge experiments were conducted to evaluate the pathogenic potential of these hen-isolated strains. Genomic DNA sequences were subjected to comparative analysis to identify virulence genes differential between newly hen-derived and previous strains.

**Results:**

Three serotypes, 1, 5, and 10, were identified using PCR and agglutination assays. Animal challenge experiments demonstrated that all three strains could induce oviduct obstruction in 30-, 60-, and 90-day-old hens. Genomic sequencing analysis revealed 18 mutated virulence genes associated with diverse virulence determinants, including type IV secretion systems (T4SSs), hemolysin, yersiniabactin (Ybt), lipooligosaccharide (LOS), lipopolysaccharide (LPS), BrkA, capsule biosynthesis, flagella, caseinolytic protease C (ClpC), FeoAB, and Vi antigens, all of which have been established as critical factors in bacterial pathogenicity.

**Conclusion:**

The findings of this study confirm an association between *R. anatipestifer* infection and reduced egg production in hens, and provide a foundation for elucidating the specific roles of virulence genes in the altered pathogenicity of *R. anatipestifer* in chickens.

## 1 Introduction

*Riemerella anatipestifer* is a Gram-negative, non-motile, rod-shaped, non-spore-forming bacterium belonging to the family *Flavobacteriaceae* (Segers et al., 1993). Based on the polymorphic diversity of capsular polysaccharide (CPS) and lipopolysaccharide (LPS) antigens, *R. anatipestifer* is divided into at least 25 distinct serotypes (Omaleki et al., 2021). Domestic ducks, including Muscovy, Cherry Valley, and Pekin ducks, are highly susceptible to infection, with mortality rates in affected flocks ranging from 5 to 75% (Swayne et al., 2020). In addition to ducks, the bacterium exhibits a broad host range, encompassing turkeys, geese, pheasants, guinea fowl, quails, and various wild waterfowl and migratory bird species (Cha et al., 2015; Swayne et al., 2020; Zhang et al., 2024). Horizontal transmission represents the primary route of dissemination, occurring mainly via the respiratory tract or through cutaneous injuries, particularly on the feet (Rubbenstroth et al., 2009). Environmental stressors such as temperature extremes and prolonged rainfall have been associated with increased disease incidence (Hao et al., 2025).

*Riemerella anatipestifer* infection has become increasingly prevalent in chicken populations in China in recent years, resulting in substantial economic losses to the poultry industry. The bacterium was first isolated in 2019 from diseased laying hens exhibiting reduced egg production and decreased hatching rates. Based on subsequent research findings, this pathogen was hypothesized to be vertically transmitted from parent birds to their offspring (Chen et al., 2024). Epidemiological investigations conducted on chicken farms between 2021 and 2024 revealed a rapid increase in *R. anatipestifer* infection rates, accompanied by an expansion in affected geographical areas (Zhang et al., 2025). Although early studies suggested that *R. anatipestifer* exhibited lower pathogenicity in chickens than ducks (Chen et al., 2024; Zhang et al., 2025), recent diagnostic reports indicate a growing number of cases in laying hens, leading to markedly reduced rates of egg production and premature culling of affected flocks.

The pathogenicity of *R. anatipestifer* is mediated by various virulence factors that facilitate its attachment to host cell surfaces, evasion of immune defense, and acquisition of nutrients (Dou et al., 2017). The M949_RS00050 gene in *R. anatipestifer* was identified as a key virulence determinant, contributing to bacterial adherence, invasion, capsular polysaccharide production, and biofilm formation. Strains with deletion of M949_RS00050 exhibited a 376-fold attenuation in virulence (Li et al., 2020). Genomic sequence analysis revealed that *R. anatipestifer* strains lacking the *phoP* gene exhibited significantly attenuated pathogenicity in ducks compared to wild-type strains. Functional assays further confirmed that this attenuation is mediated through the role of *phoP* in regulating bacterial aerotolerance (Zhang et al., 2022). Numerous virulence-associated genes have been identified across various *R. anatipestifer* strains (Wang et al., 2015; Yuan et al., 2019). Given the significant increase in the infectivity of *R. anatipestifer* in chickens, it is hypothesized that this phenotypic shift may arise from mutations in one or more virulence genes through mechanisms such as those described by Beceiro et al. (2013).

In the present study, we isolated three *R. anatipestifer* strains from laying hens exhibiting symptoms of oviduct obstruction and a decrease in rates of egg production and animal regression assay showed that all three strains could induce oviduct obstruction in hens of different ages. Based on genome comparisons of hen- and duck-derived strains, we characterized 18 virulence genes. We hypothesized that these virulence genes might be important for the altered pathogenicity of *R. anatipestifer* in chickens.

## 2 Materials and methods

### 2.1 Ethical statement

Animal husbandry and experimental protocols adhered strictly to the Guidelines for the Care and Use of Laboratory Animals established by the Poultry Institute of the Shandong Academy of Agricultural Sciences (SAAS-2025-S011).

### 2.2 Antisera, strains and animals

*Riemerella anatipestifer* antisera against serotypes 1, 2, and 7 were developed and stored by Qilu Animal Health Products Co., Ltd. using licensed vaccine strains (Production License No.: Veterinary Drug Production License (2013) 150252216), while serotypes 5 and 10 were identified by Sichuan Agricultural University (Liu et al., 2023). *R. anatipestifer* serotypes 1, 5, and 10 isolated from ducks have been thoroughly identified (Lyu et al., 2023) and stored in our laboratory (Table 1). Laying hens (30, 60 and 90-day-old) were obtained from Shandong Anchi Agriculture and Animal Husbandry Technology Group Co., Ltd. and individually housed in isolators under controlled temperature conditions with *ad libitum* access to feed and water.

**Table 1:** *R. anatipestifer* strains used in this study.

Strain	Sequence No.	Database
Hen S1	NMDCN0009F79	NMDC^a^
Hen S5	NMDCN0009F77	NMDC
Hen S10	NMDCN0009F7A	NMDC
Duck S1	NMDCN0009F78	NMDC
Duck S5	NMDCN0009BEU	NMDC
Duck S10	NMDCN0009BET	NMDC
ATCC 11845	GCA 000252855.1	NCBI
DSM 15868	GCA 000183155.1	NCBI
RA-GD	GCA 000191565.1	NCBI
RCAD0392	GCA 015291805.1	NCBI
RA-CH-2	GCA 000331695.1	NCBI

a The database was accessed via https://nmdc.cn/resource/genomics/sequence.

### 2.3 Isolation and identification of *Riemerella anatipestifer*

Laying hens from chicken farms around Shandong Province (Linyi, Yantai and Binzhou) exhibited symptoms of oviduct blockage and decreased rates of egg production were collected, and livers and oviducts were used for the isolation of *R. anatipestifer* strains using tryptic soy agar (TSA), supplemented with 4% fetal bovine serum (FBS) and maintained at 37 °C with 5% CO_2_. Colonies were confirmed using PCR analysis (primers were listed in Supplementary Table S1) (Zhu et al., 2023) and agglutination tests. Bacteria were cultured for more than 20 h, harvested, and counted for further experiments.

### 2.4 Animal experiments

Seven experimental groups were established: six infection groups and one control group. In the groups challenged with hen-isolated strains (serotypes 1, 5, and 10), laying hens aged 30 (*n* = 9), 60 (*n* = 9), and 90 (*n* = 3) days were subcutaneously inoculated into the dorsal cervical region with 0.5 mL (1 × 10^10^ CFU) of bacterial suspension. Hens aged 30 days (*n* = 9) inoculated with strains isolated from ducks (serotypes 1, 5, and 10) received the same bacterial dose. Hens in the control group aged 30 (*n* = 3), 60 (*n* = 3), and 90 days (*n* = 3) were administered an equivalent volume of phosphate-buffered saline (PBS) via the same route. All birds were monitored for 7 days post-inoculation (dpi) and clinical signs and mortality were recorded daily. At the end of the observation period, all remaining birds in the experimental groups were euthanized by immediate exsanguination following mechanical stunning and subjected to postmortem examination to evaluate anatomical lesions.

### 2.5 Genome sequence analysis

Genomic DNA was extracted using a NucleoBond HNW DNA kit (MN NucleoBond, Germany), and DNA concentration and purity were determined via Qubit4.0 (Q33226, Thermo) and Nanodrop (SMA4000, Taiwan, China). Library construction and sequencing were performed by Sangon Biotech Co. Ltd. (Shanghai, China). After sequencing, short and long reads were filtered using Fastp (v0.23.0) and Fastplong (v0.2.2), respectively, by removing low-quality adaptors and reads. The complete genome was assembled using Unicycler (v0.5.1) with default parameters, and proofread using NextPolish (v1.4.1). Following genome assembly, the quality of the genome was assessed using Benchmarking Universal Single-Copy Orthologs (BUSCO, v4.1.4) to evaluate the completeness of conserved core genes. CheckM (v1.0.12) was employed to estimate both genome completeness and contamination levels based on lineage-specific marker genes. Gene prediction was performed using NCBI PGAP to predict coding sequences (CDSs), tRNA, and rRNA. Tandemly repeated DNA motifs were identified using the TRF software (v4.09). Functional gene annotation was performed by homology-based searches using DIAMOND (v2.0.8) against multiple specialized databases.

### 2.6 Comparative genomic analysis

In pan-genome analysis, all genome sequences used were annotated or reannotated by Prokka to ensure consistent (Seemann, 2014), and pan-genome analysis was performed with Roary (Page et al., 2015). The comparisons of *R. anatipestifer* genome of hen- and duck-derived strains were accomplished using Mauve alignment (v2.4.0).

Sequences of *R. anatipestifer* strains from the NCBI database from 2010 to 2020 were selected (Table 1). Genome analysis was conducted using Roary (v3.11.2), while genome-wide single nucleotide polymorphisms (SNPs) were identified with FastTree (v2.0.0); the resulting phylogenetic tree was visualized and annotated using the iTOL platform (Letunic and Bork, 2019).

Virulence genes were identified based on functional gene annotation and compared with the virulence factor database using NCBI BLAST (v2.2.28). Virulence genes were screened from the different genes among hen- and duck-derived strains.

## 3 Results

### 3.1 Isolation of *Riemerella anatipestifer* strains from laying hens

**Figure 1:**
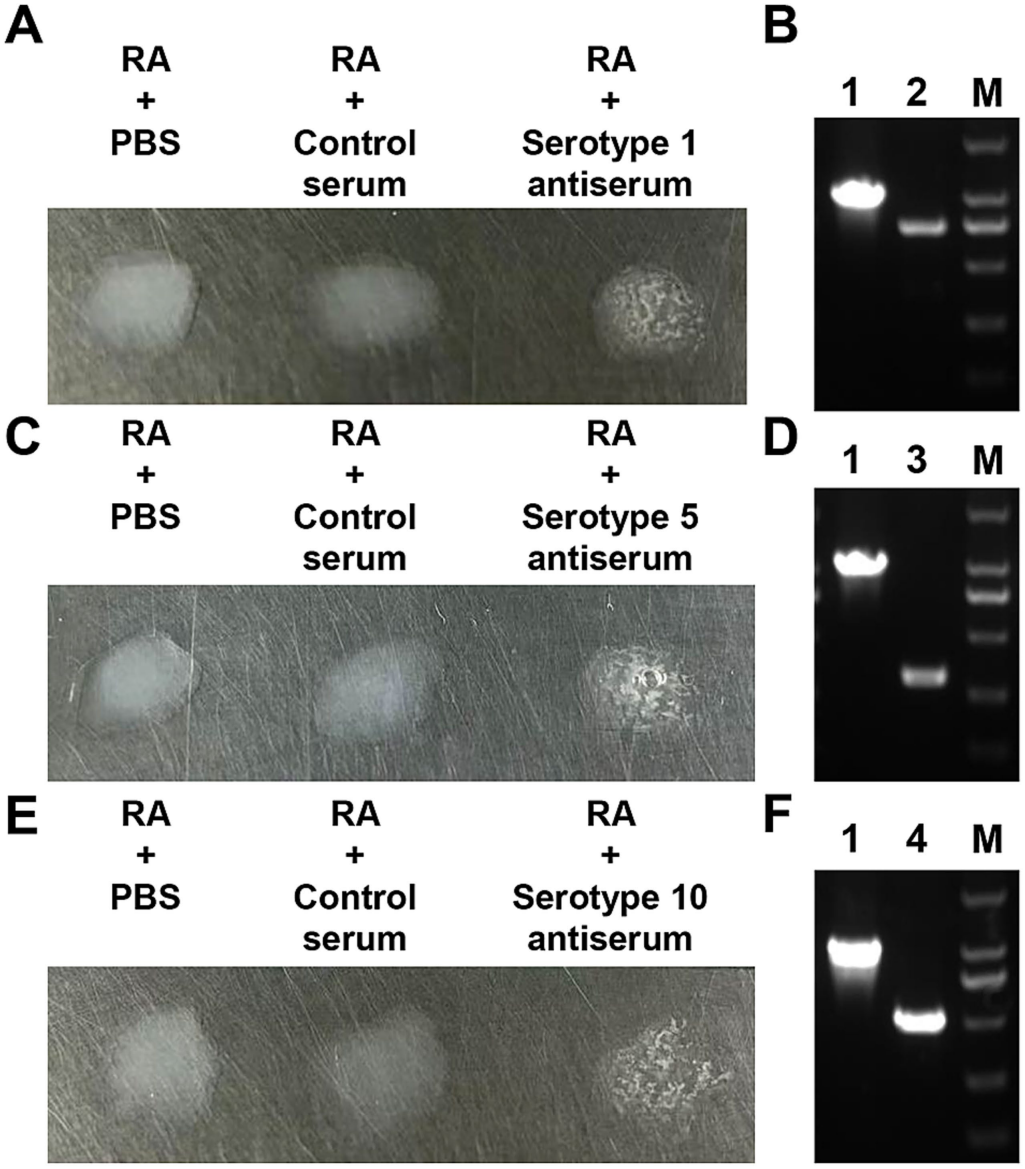
Identification of *R. anatipestifer* strains isolated from hens. **(A,C,E)** Serum agglutination assays. **(B,D,F)** PCR-based identification. Lane M corresponds to the 2000 bp DNA marker; lane 1 shows the amplicon obtained with *R. anatipestifer*-specific primers (1,112 bp); lane 2 displays the serotype 1-specific amplicon (758 bp); lane 3 corresponds to the serotype 5-specific product (308 bp); lane 4 represents the serotype 10-specific amplicon (522 bp).

*Riemerella anatipestifer* strains were isolated from diseased hens on TSA supplemented with 4% serum, and suspected colonies were identified by PCR and agglutination. The results of agglutination tests showed that three serotypes (1, 5, and 10) were present (Figures 1A,C,E). PCR amplification yielded products of expected sizes (Figures 1B,D,F), which were subsequently sequenced by Sangon Biotech Co., Ltd. (Qingdao, China). Sequence alignment of the target sequences further confirmed the strains (Supplementary Figures S1–S3).

### 3.2 Pathogenicity of *Riemerella anatipestifer* in hens

**Figure 2:**
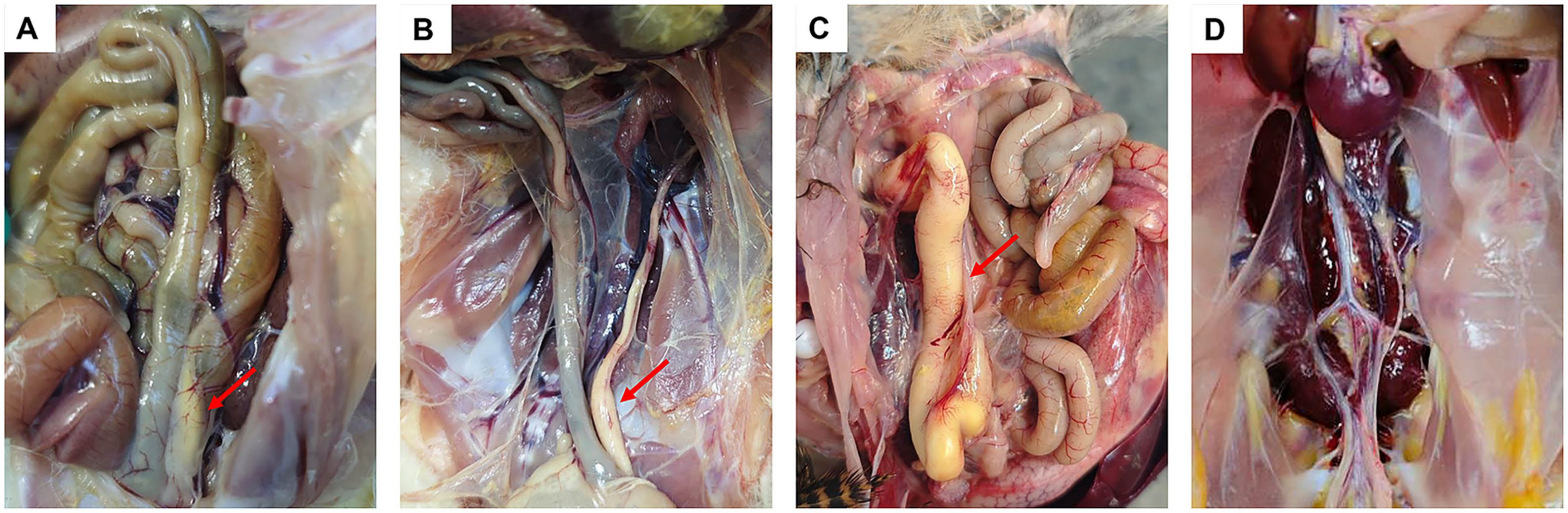
Pathological manifestations induced by *R. anatipestifer* in laying hens. Red arrows indicate sites of oviduct obstruction **(A–C)**. Laying hens (30-day-old) infected with serotypes 1, 5, and 10 of hen strain, respectively **(D)**. Laying hen (30-day-old) from the control group.

Hens in the control group exhibited no clinical signs and no mortality occurred throughout the observation period. Birds infected with strains isolated from ducks displayed symptoms, such as lethargy and reduced feed intake between 2 and 4 days-post infection, which resolved completely by 5 dpi. No mortality was observed in these groups and no pathological changes were detected on postmortem examination. In contrast, hens inoculated with newly isolated strains exhibited clinical manifestations including lameness and mortality, with two birds infected with serotype 5 and one with serotype 10 succumbing to the infection. Postmortem examination revealed consistent pathological lesions, including airsacculitis, peritonitis, hydropericardium, and oviduct obstruction, in infected birds (Figure 2). The morbidity rates among the infected groups ranged from 66.7 to 100% (Table 2).

**Table 2:** Pathogenicity experiment of *R. anatipestifer* in laying hens.

Strain (Serotype)^a^	Age (Day)	Clinical symptoms	Gross lesions	Morbidity (%)^b^
1	30	6/9 N; 3/9 L.	4/9 N; 2/9 H; 3/9 OO.	66.7
	60	9/9 N.	2/9 N; 7/9 A.	77.8
	90	3/3 N.	1/3 N; 2/3 P, OO.	66.7
5	30	3/9 N; 5/9 L; 1/9 D^c^	3/9 A; 5/9 OO.	100
	60	5/9 N; 3/9 L; 1/9 D^c^	1/9 N; 7/9 A.	88.9
	90	2/3 N; 1/3 L.	2/3 OO; 1/3 P, OO.	100
10	30	7/9 N; 2/9 L.	1/9 H; 2/9 A; 6/9 OO.	100
	60	6/9 N; 2/9 L; 1/9 D.	1/9 N; 6/9 A; 2/9 OO.	88.9
	90	3/3 N.	2/3 OO; 1/3 A, OO.	100

a *R. anatipestifer* strains of serotype 1, 5 and 10 were isolated from diseased hens.

b Morbidity was calculated based on clinical and anatomical symptoms.

c Hens were died exhibited no anatomical symptoms.

N, normal; L, lameness; D, death; H, hydropericardium; OO, oviduct obstruction; A, airsacculitis; P, peritonitis.

### 3.3 Comparative genomic analysis

**Figure 3:**
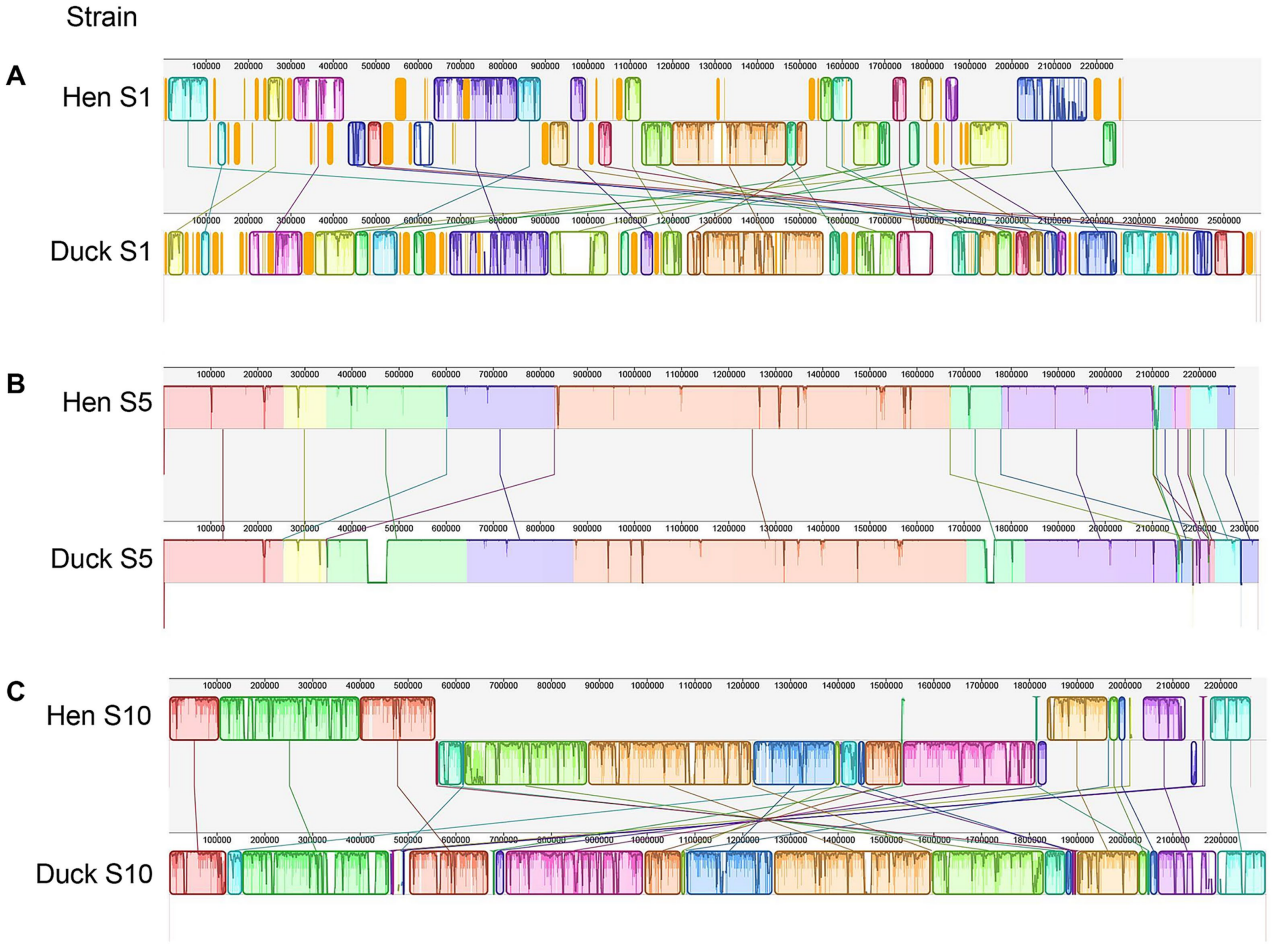
Comparative genomic analyses of strains isolated from hens and ducks. **(A–C)** Presented the comparison results between serotype 1, 5, and 10 of hen and duck strain, respectively. Each contiguously colored region is a colinear block. Lines between genomes trace each orthologous colinear block through every genome. The colinear blocks below a genome’s center line represent segments that are inverted relative to the reference genome.

Genome sequencing confirmed that *R. anatipestifer* strains isolated from hens of serotypes 1, 5, and 10 possessed genome sizes of 2,262,433 bp, 2,275,016 bp, and 2,264,455 bp, respectively. The genomes of duck strains were compared against these isolates using Mauve to assess collinearity, with alignments revealing a high degree of synteny and structural conservation between hen and duck strains (Figure 3).

**Figure 4:**
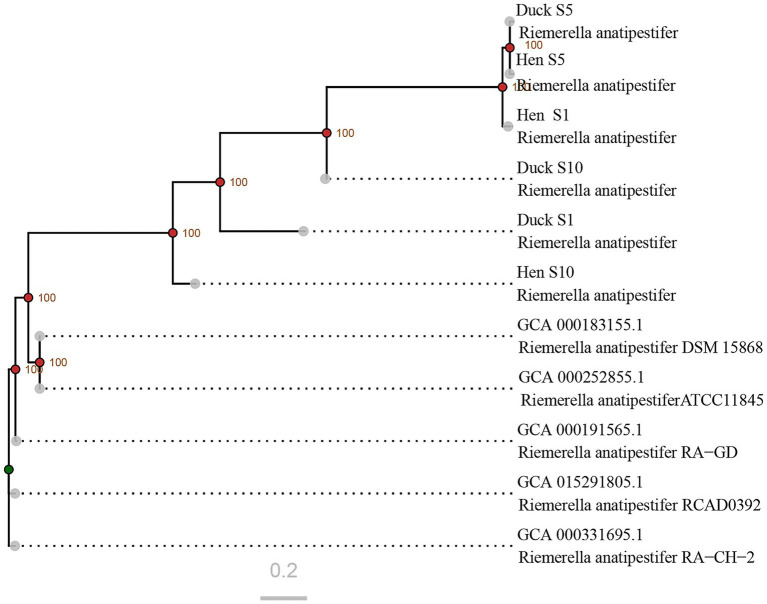
Phylogenetic relationships among *R. anatipestifer* strains based on genome-wide SNP analysis. The sequences retrieved from GenBank correspond to strains isolated between 2010 and 2020. Nodes with bootstrap values exceeding 90 are indicated by red points, whereas those with values below 70 are marked in green.

To elucidate the phylogenetic relationships among the newly *R. anatipestifer* strains isolated from hens, SNP-based phylogenetic analysis was conducted by comparing their genomic sequences with those of previously reported strains. The analysis demonstrated that the newly identified strains formed a phylogenetically distinct clade separate from historical isolates. Substantial genetic divergence was observed between hen and duck strains within serotypes 1 and 10, whereas serotype 5 strains from both hosts exhibited close phylogenetic proximity (Figure 4).

### 3.4 Characterization of virulence genes

Eighteen virulence genes were identified by functional screening in the genome of hen and duck strains. These genes are associated with diverse virulence determinants, including the type IV secretion system (T4SSs), hemolysin, yersiniabactin (Ybt), lipooligosaccharide (LOS), lipopolysaccharide (LPS), BrkA, capsule biosynthesis, flagella, caseinolytic protease C (ClpC), FeoAB, and Vi antigens. Among these, four genes were differentially identified in serotype 1 through comparative analysis of hen and duck isolates, whereas three and six genes were specific to serotypes 5 and 10, respectively. Two genes were shared between serotypes 1 and 5 as well as between serotypes 1 and 10, and one common gene was observed in both serotypes 5 and 10 (Table 3).

**Table 3:** Characterization of different virulence genes between hen and duck strains of *R. anatipestifer.*

Gene name	Virulence factor name	Serotype	Description of gene
*bepA*	T4SSs	1	Establishment of infection to endothelial cells; colonization of endothelial cells; mediating the intracellular delivery of effector proteins.
*hlyB*	Hemolysin	1	Cytotoxic to many types of cells; stimulating the release of IL-1β and TNF, membrane-damaging.
*wbaP/rfbP*	LOS	1	Initiation enzymes for O-polysaccharide formation.
*ybtS*	Ybt	1	High affinity for ferric iron, and uptake iron from host cells.
*lpxA*	LOS	1, 5	Participant in the pathway of lipid A biosynthesis.
*gtrB*	LPS	1, 5	Required for resistance to host defense and for the intracellular spread
*gluP*	LPS	1, 10	Lower immunobiological activities; mediating lectin-like interaction with laminin; adherence to host cells.
*brkB*	Brk	1, 10	Outer membrane protein that mediates adherence and resists complement.
*cpsl*	Capsule	5	Contributes to host immune evasion.
*motB*	Flagella	5	Enhance the invasion capacity.
*clpC*	ClpC	5	Promoting the escape from the phagosome of macrophages; participating adhesion and invasion to host cells.
*feoB*	FeoAB	5, 10	Contribute the uptake of iron and ferrous iron.
*bplA*	LPS	10	Prevent clearance of the bacteria; protect the bacterium from complement-mediated cell lysis.
*cpsB*	Capsule	10	Contributes to evasion from host immune.
*cpsN*	Capsule	10	Prevent the activation of the alternative complement pathway; inhibit complement-mediated phagocytosis.
*glf*	Capsule	10	Participate in bacterial survival and the evasion of host immune response; important for virulence and adhesion.
*kdsB*	LOS	10	Participates in LPS biosynthesis.
*tviC*	Vi antigen	10	Prevent antibody-mediated opsonization; increase resistance to host peroxide and resistance to complement activation.

## 4 Discussion

China’s poultry industry has undergone rapid development, encompassing the production of chickens, ducks, geese, and other poultry species. By 2025, the total number of slaughtered poultry will exceed 2.2 billion. In intensive farming regions, chicken, duck, and goose farms are often situated in close proximity, with some operations involving mixed farming of chickens and ducks. This practice has facilitated the cross-species transmission of pathogens, leading to cross-infections between chicken and duck infectious agents. Tembusu virus (TMUV) infections have recently been reported in chickens and geese (Yang et al., 2023; Zhou et al., 2023); previously the virus has been primarily detected in ducks. *R. anatipestifer* has historically been considered a pathogen that exclusively affects ducks (Ke et al., 2022). However, an increasing number of *R. anatipestifer* strains have been isolated from laying hens that exhibit decreased egg production (Figure 1), suggesting that this bacterium may be responsible for this symptom. Results from the animal challenge experiments demonstrated that infection with *R. anatipestifer* induced the typical symptoms of oviduct obstruction (Figure 2), indicating a direct association between bacterial infection and reduced egg production in laying hens.

The pathogenicity of bacteria is primarily determined by the virulence genes (Yuan et al., 2019; Zhu et al., 2019). To adapt to commensal niches, bacterial virulence genes are continually shaped by diverse host and environmental factors (Quereda et al., 2021). In 2019, integrative conjugative elements were first identified in *R. anatipestifer*, which contribute to genetic diversity, evolution, adaptation, and virulence (Zhu et al., 2019). Phylogenetic analysis revealed that the newly identified strains formed a clade distinct from the previous isolates (Figure 4), suggesting ongoing genetic evolution in *R. anatipestifer*. However, genetic divergences between newly isolated hen strains and duck strains within each serotype used in this experiment were found to be relatively limited, particularly among serotype 5 strains, indicating that genome differences between these strains were small. Thus, we speculated that one or more key virulence genes within the genome might contribute to the alterations in pathogenicity via some known or unknown pathway, which would be evaluated in future studies.

Based on the comparative genomic analysis of strains isolated from hens and ducks, we identified 18 virulence genes associated with diverse virulence determinants, including T4SSs, hemolysin, Ybt, LOS, LPS, BrkA, capsule biosynthesis, flagella, ClpC, FeoAB, and Vi antigens (Table 2). LPS is a major structural component of the bacterial membrane and participates in the stimulation of innate immune responses (Kalynych et al., 2014). Similarly, capsules are critical for the virulence of invasive bacteria, as they facilitate the evasion of molecular recognition and phagocytic clearance through multiple mechanisms (Brissac et al., 2021; An et al., 2022). In addition, flagella contribute to bacterial motility, which is a key virulence attribute of many pathogenic species (Nakamura and Minamino, 2019). Similarly, LOS is densely expressed on bacterial surfaces, and serves as a prominent target for adaptive immune responses (Gulati et al., 2019). T4SSs in many bacteria assemble surface structures, such as conjugative pili or adhesins, thereby promoting attachment and biofilm formation (Costa et al., 2024). In contrast, Ybt enhances bacterial virulence by binding and sequestering metal ions (Price et al., 2021). Hemolysin induces programmed necrosis by disrupting mitochondrial dynamics, as demonstrated in *E. coli* (Lu et al., 2018). The Vi antigen aids in immune evasion by reducing complement C3b deposition and impairing the macrophage-mediated clearance of *S. typhi* (Crawford et al., 2013). Furthermore, ClpC is essential for normal developmental progression in *Chlamydia* (Pan et al., 2023), while FeoAB supports ferrous iron acquisition in *R. anatipestifer* and is critical for bacterial colonization (Huang et al., 2023). Collectively, these virulence factors are well established as major determinants of bacterial pathogenicity; however, their specific roles in the altered pathogenicity of *R. anatipestifer* in chickens require further investigation.

## 5 Conclusion

In this study, three *R. anatipestifer* strains of serotypes 1, 5, and 10 were isolated from laying hens that exhibited clinical signs of oviduct obstruction and reduced egg production. Animal challenge experiments demonstrated that all three strains induced similar clinical manifestations in hens of different ages, suggesting that *R. anatipestifer* infection may be a major causative factor for the decline in egg production. To investigate the mechanisms underlying this shift in pathogenicity, we sequenced and compared the genomes of hen and duck strains. Through comprehensive genomic analysis, we identified 18 mutated virulence genes associated with diverse virulence determinants, including T4SSs, hemolysin, Ybt, LOS, LPS, BrkA, capsule biosynthesis, flagella, ClpC, FeoAB, and Vi antigens, all of which are major determinants of bacterial pathogenicity. Further studies are needed to elucidate the specific roles of these genes in the altered pathogenicity of *R. anatipestifer* in chickens.

## Data Availability

The original contributions presented in the study are publicly available. This data can be found in the National Microbiology Data Center (https://nmdc.cn/resource/genomics/sequence) with the accession numbers in Table 1.
