# Supplementary material for: Genome analysis screening virulence genes for the altered pathogenicity of Riemerella anatipestifer in hens
Source: Front Microbiol. 2025 Dec 10;16:1705927. doi: 10.3389/fmicb.2025.1705927 (PMC12727888; doi:10.3389/fmicb.2025.1705927)
Supplement: Supplementary file 1 [file Data_Sheet_1.docx]

| Table S1 Primers used for the identification of *R. anatipestifer* | | |
| --- | --- | --- |
| Name | Product (bp) | Sequence (5’—3’) |
| Ra F | 1112 | GCAGAGGGACAAGCTCCTTT |
| Ra R |  | TGTGCCAACCAATATTGAGCC |
| Ra S1 F | 758 | TTCAAGGAGGGGGAGGGTTA |
| Ra S1 R |  | TGCAACAACAGCTCCACTTG |
| Ra S5 F | 308 | TCGGGAATACTTGGCGCAAT |
| Ra S5 R |  | GCAAAAACACCCGAGGATGC |
| Ra S10 F | 522 | CAGCGGATAGGCTGTATCCAT |
| Ra S10 R |  | TAGGAGCACCAATATCCCCGA |
| Ra F/R were used to identify *R. anatipestifer* strains; Ra S1 F/R were used to identify *R. anatipestifer* serotype 1 strains; Ra S5 F/R were used to identify *R. anatipestifer* serotype 5 strains; Ra S10 F/R were used to identify *R. anatipestifer*  serotype 10 strains. | | |

**Figure S1** The sequence analysis of serotype 1 of hen strain. A, the alignment of *R. anatipestifer* sequence with sequence amplified with Ra primers. B, the alignment of *R. anatipestifer* S1 sequence with sequence amplified with Ra S1 primers.

**
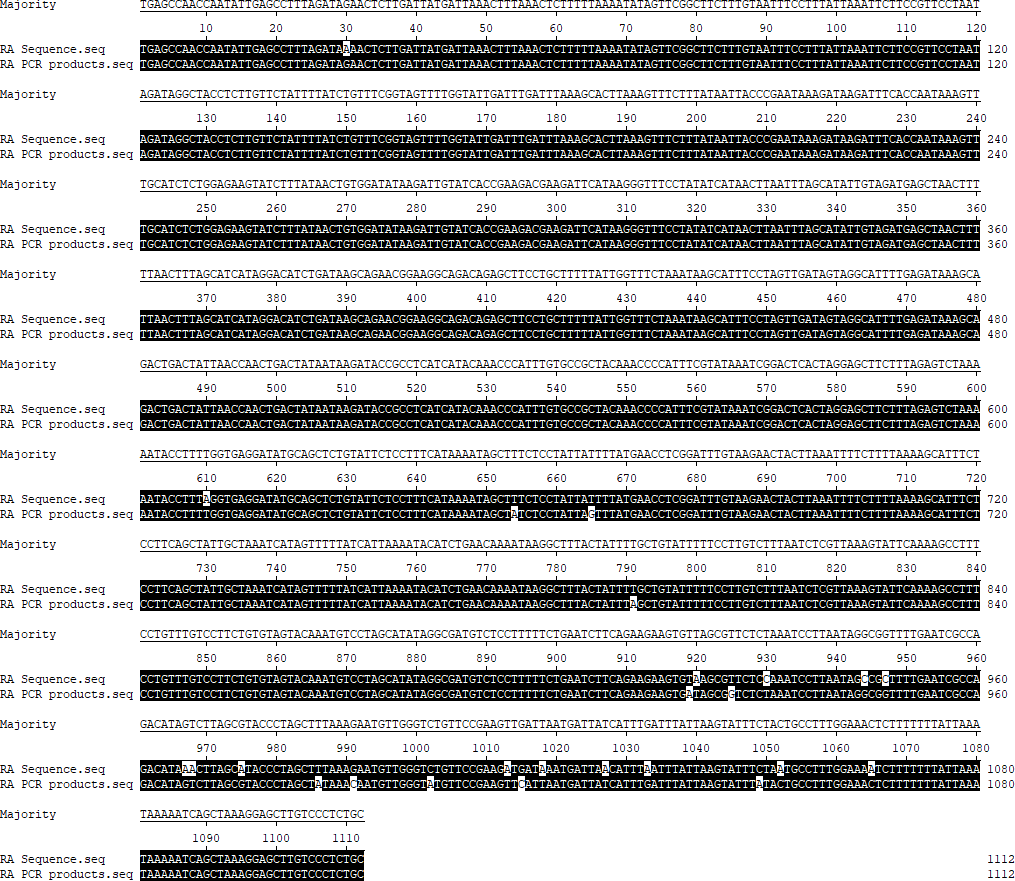

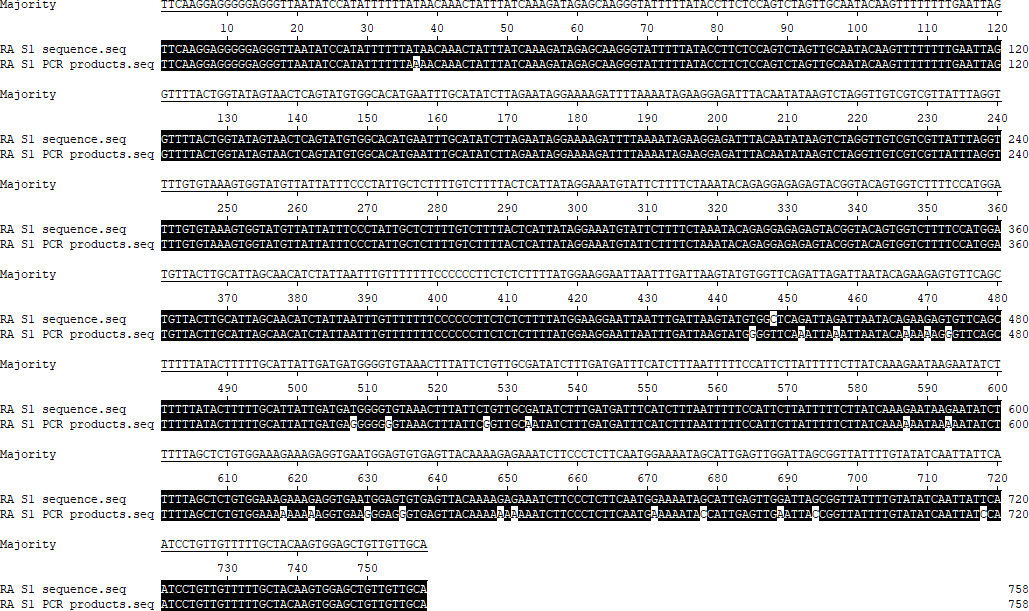
A B**

**Figure S2** The sequence analysis of serotype 5 of hen strain. A, the alignment of *R. anatipestifer* sequence with sequence amplified with Ra primers. B, the alignment of *R. anatipestifer* S5 sequence with sequence amplified with Ra S5 primers.

**
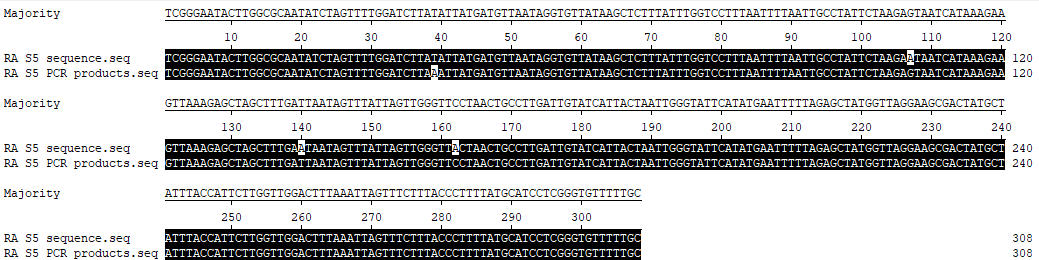

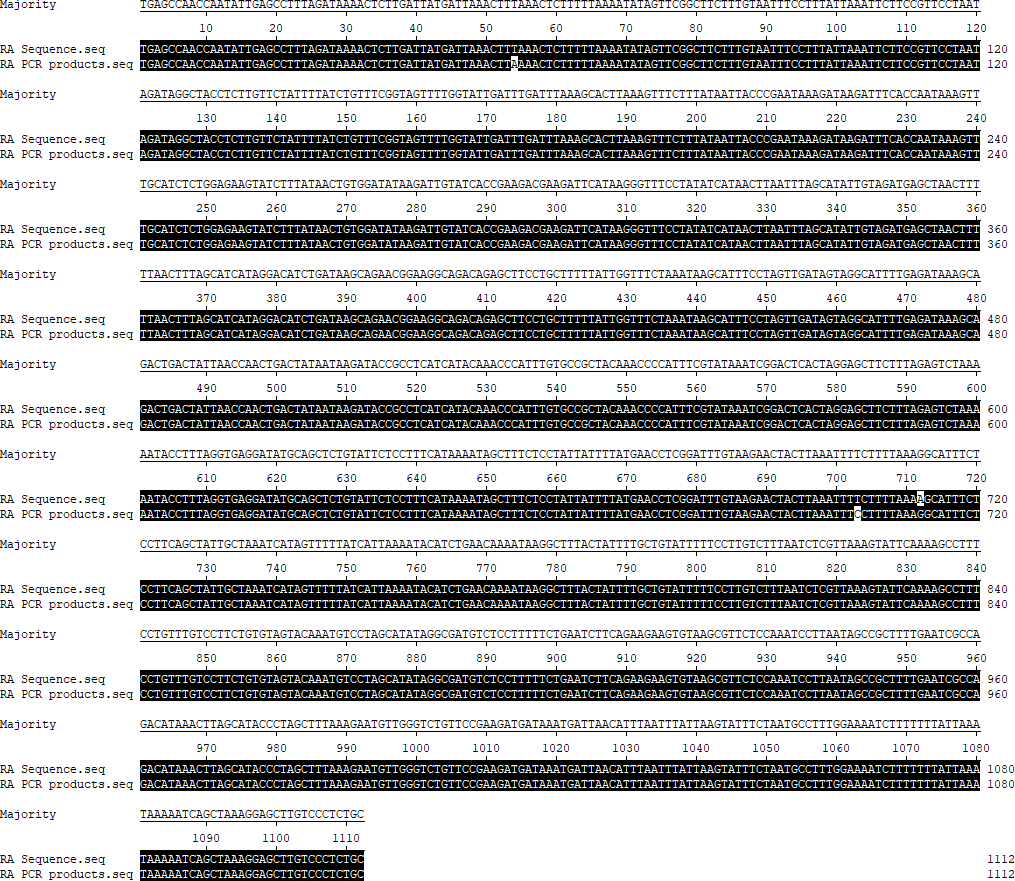
A B**

**Figure S3** The sequence analysis of serotype 10 of hen strain. A, the alignment of *R. anatipestifer* sequence with sequence amplified with Ra primers. B, the alignment of *R. anatipestifer* S10 sequence with sequence amplified with Ra S5 primers.

**
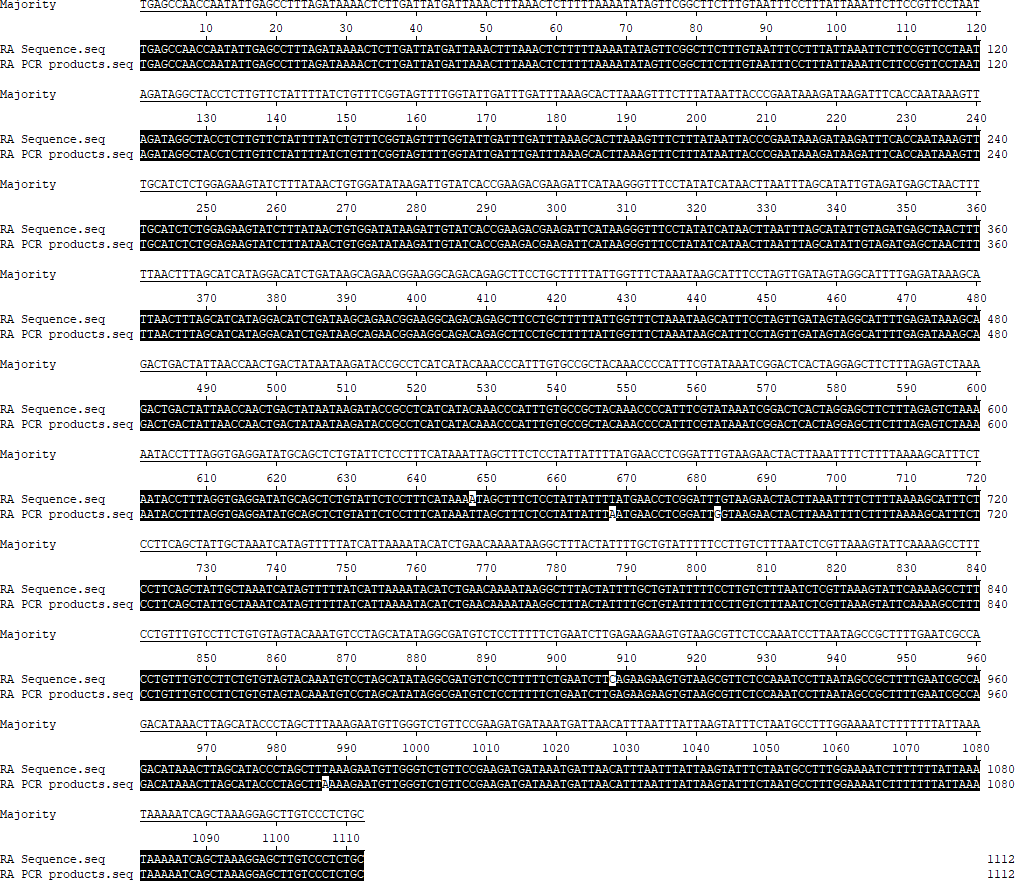

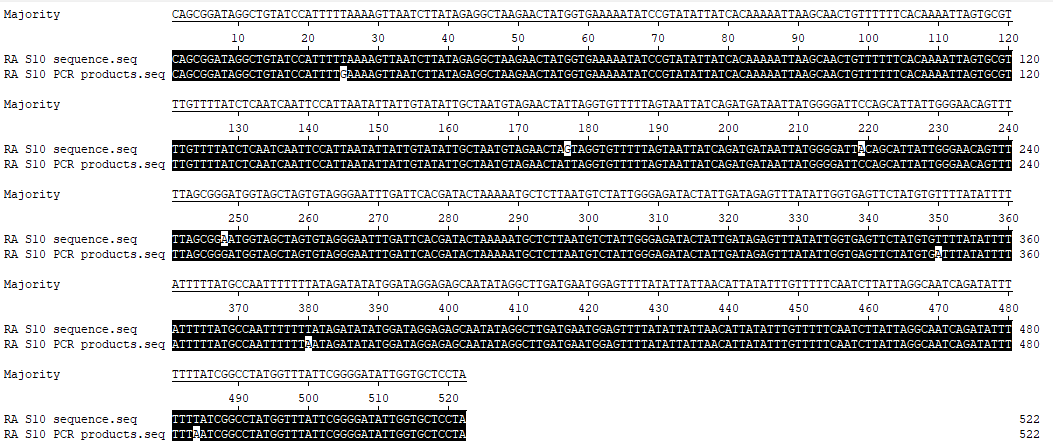
A B**
